# Eosinophilic Cells as a Distinct Morphological Feature in *BRAF^V600E^*-Mutated Ovarian Serous Borderline Tumors

**DOI:** 10.3390/diagnostics15121479

**Published:** 2025-06-11

**Authors:** Alina Badlaeva, Anna Tregubova, Aleksandra Asaturova, Gennady Sukhikh

**Affiliations:** 1 National Medical Research Center for Obstetrics, Gynecology and Perinatology Named After Academician V.I. Kulakov of the Ministry of Health of Russia, Bldg. 4, Oparina Street, 117513 Moscow, Russia; a_badlaeva@oparina4.ru (A.B.); a_tregubova@oparina4.ru (A.T.); sukhikh@oparina4.ru (G.S.); 2 Department of Pathological Anatomy and Clinical Pathological Anatomy, Pirogov Russian National Research Medical University, Bldg. 1, Ostrovitianov Street, 117997 Moscow, Russia

**Keywords:** serous borderline tumor, BRAF, KRAS, NRAS, eosinophilic cells, oncogene-induced senescence, interobserver reproducibility

## Abstract

**Background/Objectives**: According to recent reports, the *BRAF^V600E^* mutation in serous borderline tumors (SBTs) plays a protective role against progression to low-grade serous carcinoma through oncogene-induced senescence. One consequence of this is the appearance of eosinophilic cells (ECs). The aim of the current study was to determine the interobserver reproducibility of ECs and their predictive significance for the detection of the *BRAF^V600E^* mutation in SBTs. **Methods**: The study was conducted using 63 cases of ovarian SBTs. Three gynecological pathologists, blinded to each tumor’s mutation status, assessed the presence of ECs. Immunohistochemical staining with p16 and Ki-67 was performed to validate ECs. Mutational analysis was carried out using targeted NGS. **Results**: Genetic analysis revealed 30 *BRAF*-mutated, 1 *NRAS*-mutated, and 9 *KRAS*-mutated SBTs. ECs were identified by the majority of pathologists (two or three) in 78% of the *BRAF^V600E^*-mutated and 11% of the wild-type tumors with other mutations (*p* < 0.0001). The interobserver reproducibility of the presence of ECs was substantial (κ = 0.66). ECs validated with p16/Ki-67 were identified in 92.6% of the *BRAF^V600E^*-mutated and in 13.8% of the wild-type tumors with other mutations (*p* < 0.0001). For the ECs identified by the majority of pathologists, the sensitivity and specificity when predicting the *BRAF^V600E^* mutation were 77.8% and 88.9%, respectively. For the ECs validated with p16/Ki-67, the sensitivity and specificity when predicting the *BRAF^V600E^* mutation were 95.3% and 90.5%, respectively. **Conclusions**: Overall, these results suggest that ECs in SBTs have potential association with the *BRAF^V600E^* mutation.

## 1. Introduction

Serous borderline tumor (SBT) of the ovary is now regarded as a type of non-invasive neoplasm with low malignant potential and an indolent course [1]. Histologically, this tumor is characterized by multiple papillae with extensive and complex hierarchical branching. Its frequency is between 9 and 15% among all serous ovarian tumors. SBT is usually detected at an early stage (FIGO stage I) and has a high survival rate [1,2].

There are a number of longitudinal studies in which risk factors for subsequent low-grade serous carcinoma (LGSC) have been described [3,4,5]. These include ovarian surface involvement, bilateral tumor localization, stage > I according to FIGO, residual tumor, and a micropapillary growth pattern [3]. However, despite the absence of the above-mentioned signs of progression, low-grade serous carcinoma can still occur several years after SBT [3].

Over the past decades, numerous researchers have attempted to describe the role of the molecular events responsible for the onset and progression of SBT [6,7]. It has been clearly shown that the majority of SBTs have mutations in the MAPK signaling pathway (e.g., BRAF or KRAS). While tumors with a *KRAS* mutation are more likely to be associated with recurrence and progression to carcinoma, a *BRAF* mutation is a significant prognostic factor for a favorable course of the disease [6,7].

Recent data suggest that there may be a link between *BRAF* mutations and a specific histological feature—eosinophilic cells (ECs) [8]. The latter can be described as senescent cells with abundant eosinophilic cytoplasm. ECs result from oncogene-induced senescence (OIS) via the activation of oncogenes (RAF and RAF families are the most frequently described) [9]. This leads to upregulation of E2F7, a transcriptional decrease of the regulatory subunit of ribonucleotide reductase M2 (RRM2), and, subsequently, to low deoxyribonucleotide triphosphate (dNTP) levels, resulting in DNA damage, replication stress, and cell growth arrest [9].

Taken together, the above results suggest that *BRAF* mutations appear to prevent consistent progression from SBT to LGSC, and that ECs may be a marker for these genetic drivers. However, to date, there has been little discussion of reproducibility in the identification of ECs in SBTs among pathologists. For instance, the ECs associated with the *BRAF^V600E^* mutation and their interobserver reproducibility among pathologists have already been investigated in the study by Chui [10], so the current study represents an “incremental” innovation compared to the aforementioned work.

Therefore, the aim of the current study was to determine the interobserver reproducibility of ECs and their predictive significance for the detection of the *BRAF^V600E^* mutation in SBTs.

## 2. Materials and Methods

The data for this study were collected from 63 consecutive cases of ovarian SBTs in eligible patients aged 15 to 79 years who underwent surgery at a single center during the calendar years 2017–2024. Hematoxylin and eosin (H&E) slides were obtained from formalin-fixed paraffin-embedded (FFPE) specimens that were routinely prepared and collected from the biosample repository of the Research Center for Obstetrics, Gynecology, and Perinatology (Moscow, Russia). The inclusion criteria were as follows: samples with ovarian SBTs (including micropapillary patterns) and representative samples (more than 20% tumor tissue). The exclusion criteria were as follows: residual tumors and inadequate or inappropriate samples (less than 20% tumor tissue).

Only 2 cases of SBTs with a micropapillary pattern were included in our series. In 8 of the 63 cases, incomplete cytoreductive surgery was performed because no frozen section was available, so no surgical tumor staging was performed in these cases.

**Figure 1:**
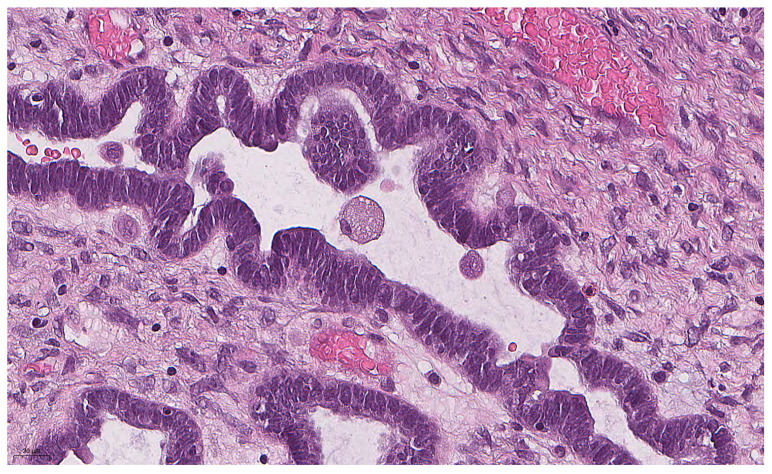
Eosinophilic cell with abundant eosinophilic cytoplasm and small nucleus in SBT (previously unpublished, original photo) with hematoxylin and eosin staining (magnification 400×).

The histological slides were independently assessed by three gynecologic pathologists to confirm the diagnosis and evaluate the presence of ECs as a hallmark of the *BRAF^V600E^* mutation. According to Chui [10], ECs are defined as follows: easily detectable round cells with abundant dense/glassy eosinophilic cytoplasm occupying at least 50% of the cell area at 100× magnification (Figure 1). All pathologists were trained to recognize ECs using an interobserver reproducibility study training set (http://BRAFsbt.wordpress.com, accessed on 16 December 2024). A binary score (present or absent) was used to assess the extent of ECs, with 0 indicating the complete absence of ECs and 1 indicating the presence of at least a single EC. All cases were analyzed blindly in terms of mutation status.

**Figure 2:**
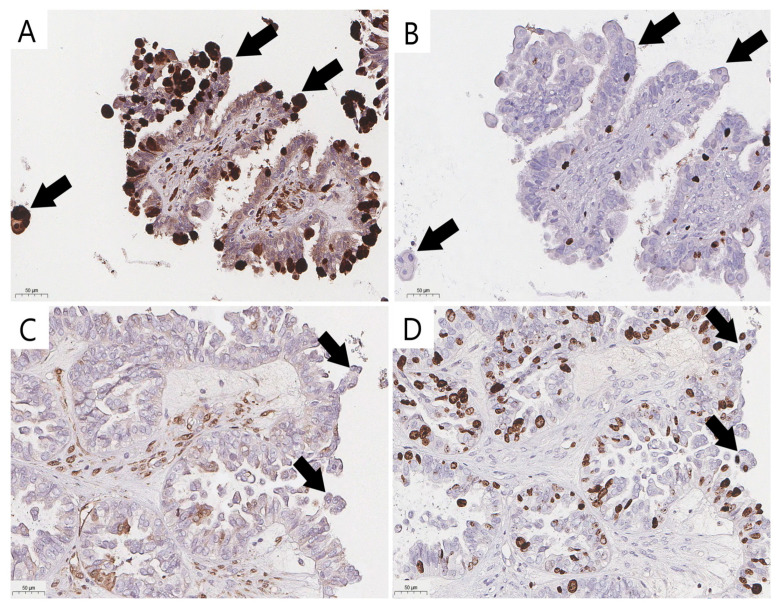
Validation of eosinophilic cells and their mimics (arrow) in ovarian SBTs (previously unpublished, original photos). Strong nuclear and cytoplasmic staining of p16 in senescent eosinophilic cells (**A**) and lack of nuclear expression of proliferative activity marker Ki-67 (**B**) in senescent eosinophilic cells; absence of strong nuclear and cytoplasmic staining of p16 (**C**) and positive expression of Ki-67 (**D**) in cells that could be misidentified as bonafide ECs. Images feature immunohistochemical staining (magnification 200×).

Immunohistochemical staining (IHC) with the antibodies p16 (CINtec p16, ready-to-use, Ventana, AZ, USA) and Ki-67 (clone 30-9, ready-to-use, Ventana, AZ, USA) was performed with the VENTANA UltraView DAB IHC Detection Kit (Ventana Medical-Systems, Oro Valley, AZ, USA) and a BenchMark XT automated immunostainer (Ventana Medical-Systems, Oro Valley, AZ, USA). To validate the reproducibility, a consensus diagnosis was made using true senescent ECs verified with p16 and Ki-67 IHC staining (Figure 2A,B) to distinguish them from their mimics with overlapping cytological features (Figure 2C,D). Positive staining to determine the senescent immunophenotype was defined as strong diffuse nuclear-cytoplasmic staining for p16 and the absence of Ki-67 expression (Figure 2A,B).

Mutation analysis was performed by the targeted high-throughput next-generation sequencing (NGS) of amplicons on a FASTASeq300 sequencer (Gene-Mind Bioscience, Shenzhen, China). DNA was isolated from FFPE tumor material using the QIAamp DNA FFPE tissue kit (Qiagen, Germantown, MD, USA) on columns according to the standard protocol recommended by the manufacturer. Specific primers for KRAS (exons 2, 3, and 4), NRAS (exons 2, 3, and 4) and BRAF (exon 15) were used for genetic analysis, which were selected using the Lasergene 17.6 software suite (DNASTAR software package, Madison, WI, USA). Library preparation included DNA tagmentation with the attachment of sequencing adapters, amplification of the tagmented DNA, and purification of PCR products using magnetic beads. For paired-end reading, MiSeq Reagent Kit v2 reagents (Illumina, San Diego, CA, USA) were used with a maximum read DNA fragment length of 300 nucleotides, which provided high-quality full-length reads.

Statistical analyses were performed using GraphPad Prism 9.3.1 (Dotmatics, Boston, MA, USA). Comparisons between the groups according to mutations were made using Kruskal–Wallis and chi-square tests. To measure interobserver agreement during the assessment of ECs, Fleiss’s kappa and Cohen’s kappa were calculated, where a kappa value of 0 to 0.4 indicated no or minimal agreement, 0.4 to 0.6 moderate agreement, 0.6 to 0.8 substantial agreement, and 0.8 to 1 near-perfect agreement. In order to determine the accuracy of the EC identification, their specificity, sensitivity, positive predictive value (PPV), and negative predictive value (NPV) were estimated. The following formulae were used:

$$Specificity=\frac{true\;negative}{true\;negative+false\;positive}\times 100$$

$$Sensitivity=\frac{true\;positive}{true\;positive+false\;negative}\times 100$$

$$PPV=\frac{true\;positive}{true\;positive+false\;positive}\times 100$$

$$NPV=\frac{true\;negative}{true\;negative+false\;negative}\times 100.$$

Statistical testing results were significant at the *p* = 0.05 level.

## 3. Results

The genetic analysis revealed that around half of the SBTs (30 of 63 cases, 47.6%) had a *BRAF* mutation. The *BRAF* mutations consisted of 27 cases of the usual T to A substitution at position c.1799 (c.1799T>A) and 3 cases of A to G substitution at position c.1781 (c.1781A>G) in exon 15 of the coding DNA. *KRAS* mutations occurred in 9 of the 63 cases (14.3%). One *NRAS* mutation in SBT was revealed, consisting of p.Gln61Arg (c.182A>G). All mutation variants are available in Table 1. Double mutations were not observed.

As Table 2 shows, the patients in the BRAF-mutated group were younger than those in the other groups. Nevertheless, the statistical tests showed no significant differences between the groups (*p* = 0.16).

In eight cases, no information on the FIGO stage was available due to incomplete surgical intervention. Thus, the results of the study showed that of the 55 serous borderline tumors, a FIGO stage > I was found in 11% of the *BRAF*-mutated tumors (2—V600E and 1—Asp594Gly), in 66.7% of the *KRAS*-mutated tumors, and in 50.0% of the wild-type tumors. Consequently, *BRAF*-mutated SBTs were less likely to have noninvasive implants in the omentum and peritoneum compared to other tumors (*p* = 0.004). A short-term follow-up of 3 years was available for 15 patients, 3 of whom had tumor recurrence (all cases in the *BRAF*-mutated group). Of the 10 cases with a mid-term follow-up (5 years), there was 1 case of recurrence in the *KRAS*-mutated group.

**Figure 3:**
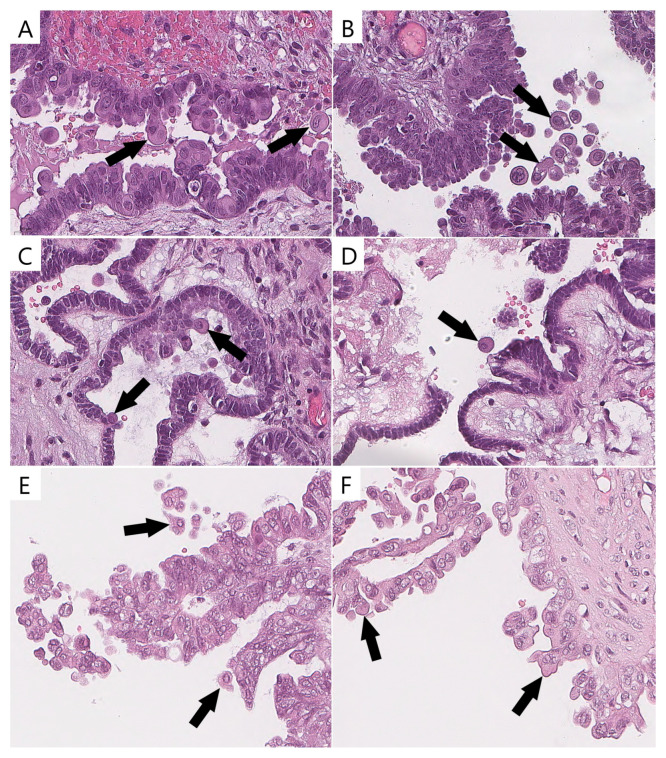
Eosinophilic cells in *BRAF^V600E^*-mutated tumors and their mimics in wild-type cases (previously unpublished, original photos). Bonafide eosinophilic cells with abundant dense/glassy eosinophilic cytoplasm occupying at least 50% of cell area (arrow, (**A**–**D**)); epithelial cells detaching from tumor surface and resembling eosinophilic cells (arrow, (**E**,**F**)). Images feature hematoxylin and eosin staining (magnification 400×).

ECs were identified by the majority of pathologists (two or three) in 78% (21/27) of the *BRAF^V600E^*-mutated (Figure 3) tumors and in 11% (4/36) of the wild-type tumors with other mutations (*p* < 0.0001). ECs validated with p16/Ki-67 were identified in 92.6% (25/27) of the *BRAF^V600E^*-mutated tumors and in 13.8% (5/36) of the wild-type tumors with other mutations (*p* < 0.0001).

The interobserver reproducibility of the presence of ECs among the pathologists was substantial (κ = 0.66, 95% confidence interval, CI 0.659–0.668). A possible explanation for this result could be the presence of stratified and tufted cells in a number of wild-type SBTs, which could be misidentified as bonafide ECs (Figure 3). The Cohen’s kappa results are shown in Table 3.

The interobserver variation of the ECs identified by the three pathologists and the ECs validated with p16/Ki-67 staining and sorted by *BRAF* mutation status are shown in Appendix A (Table A1).

For the ECs identified by the majority of pathologists (two or three), the sensitivity and specificity when predicting the *BRAF^V600E^* mutation were 77.8% and 88.9%, respectively. The positive predictive value was 84%, and the negative predictive value was 84.2%. For the ECs validated with p16/Ki-67, the sensitivity and specificity of the prediction of the *BRAF^V600E^* mutation were 95.3% and 90.5%, respectively. No bonafide ECs were detected in the three samples with the *BRAF^Asp594Gly^* mutation.

## 4. Discussion

SBT is an indolent neoplasm with low malignant potential that represents the most common histological subtype among all ovarian borderline tumors [1,2]. Nevertheless, SBT can lead to LGSC in a small percentage of cases [3,4,5].

*BRAF* and *KRAS* mutations are important driving factors in SBT tumorigenesis and are associated with the MAPK signaling pathway and uncontrolled proliferation [6,7]. Thus, SBTs with *KRAS* mutations can lead to LGSC [7]. Recent cases reported by Chui et al. (2019) also support the hypothesis that *KRAS* mutations in SBTs are associated with a higher frequency of subsequent LGSC [3]. In this study, SBT/carcinoma pairs showed concordant profiles in 56% of *KRAS*-mutated tumor cases, 31% of wild-type tumor cases, and only 13% of *BRAF*-mutated tumor cases [3]. It is, therefore, assumed that *BRAF* mutations play a protective role in the progression to LGSC through the upregulation of tumor suppressor genes and OIS [6]. One consequence of the latter is the occurrence of ECs with a senescent phenotype.

At present, it is not yet fully understood how ECs acquire their form factor. The cell enlargement associated with senescence and the abundant eosinophilic cytoplasm are probably caused by several mechanisms. Firstly, this could be due to reduced activity of proteasomal peptidases and disruption of the ubiquitin-proteasome system. As a result, there is an excessive accumulation of oxidized and ubiquitinated dysfunctional proteins in the cytoplasm [11,12]. Secondly, an increasing bioenergetic deficiency can lead to a shift in mitochondrial dynamics towards fusion and the formation of megamitochondria. Moreover, senescent cells exhibit morphological abnormalities and lose monolayer integrity, which may be caused by downregulation of intercellular junctions [13,14].

What is not yet clear is how *BRAF*-mutated SBTs with OIS overcome senescence and cell growth arrest and still progress to LGSC. It has been suggested that additional mutations in SBTs may contribute to the tumor’s progression. Zeppernick (2015) provides an in-depth analysis of this paradox that shows the potential role of ch1p36 and ch9p21 deletions in the progression of SBT to LGSC [8]. Among the several potential tumor suppressors found in the ch1p36 region is miR-34a, which is a direct p53 target and is required for the DNA damage response [8]. Similarly, three tumor suppressor proteins that block cyclin-dependent kinase (p14 (Arf), p16, and p15) are encoded by the ch9p21 region and correspond to the CDKN2A/B locus [8,15,16].

The evidence presented so far supports the idea that the bypass of OIS and the subsequent progression of the tumor may be related to its metabolism. Following this concept, Aird et al. (2013) demonstrated that exogenous nucleosides are sufficient to overcome OIS-associated cell growth arrest [9]. This view is supported by Zeppernick (2015), who wrote that the *BRAF* mutation shows upregulation of glucose transporter-1 (GLUT1), which is an essential surface protein that leads to the increase in glucose metabolism required to overcome OIS-associated senescence and tumor progression [8].

This study confirms the potential association between BRAF-mutated SBTs and senescent cells with abundant dense eosinophilic cytoplasm. These findings match those observed in earlier studies [6,8,10,17]. In summary, the obtained results show that the histomorphological assessment of ECs by the majority of pathologists had substantial sensitivity and specificity (77.8% and 88.9%, respectively) during the prediction of the *BRAF^V600E^* mutation in SBTs. This finding is in agreement with Chui’s (2023) and Turashvili’s (2018) findings, which showed that the median sensitivity and specificity for ECs to identify SBTs with the abovementioned mutation were 67% and 95%, and 92% and 70%, respectively [10,17]. This is also consistent with our previous observation, which showed that ECs have significant sensitivity and specificity (78.9% and 91.3%, respectively) when predicting the *BRAF^V600E^* mutation [18]. The current study also found that validation of ECs with p16 and Ki-67 IHC staining could improve their sensitivity and specificity during the prediction of the *BRAF^V600E^* mutation. Interestingly, in this study, no true ECs were detected in the three cases with *BRAF^Asp594Gly^*, supporting the idea that ECs are only characteristic of the V600E mutation.

In the present study, the interobserver agreement according to Fleiss’s kappa was 0.66. Overall, this result indicates substantial reproducibility in the detection of ECs between pathologists. These results are consistent with those of our previous study, in which the inter-rater agreement between three observers was 0.7 [18]. However, they differ from the study published by Chui (2023), in which Fleiss’s kappa was 0.41 [10]. There are several possible explanations for these results. These differences could be related to a semi quantitative assessment of ECs and the fact that more observers participated in Chui’s study, while a binary assessment was used in this study.

The discordant results in interobserver reproducibility could be due to another morphological mimic of ECs: tuft-like or hobnail-shaped tumor cells lining the papillae of SBTs. However, true ECs are more rounded, with abundant eosinophilic cytoplasm, lacking cilia and mitotic activity [8,10,17].

The lower interobserver reproducibility could also be due to morphological mimics such as micropapillary SBTs; this tumor pattern was observed in only two cases in the present study, while 12 specimens with micropapillary features occurred in Chui’s research [10]. In the current study, one reviewer found ECs in one case of micropapillary SBTs without mutations, but these were not observed in this tumor by the majority of pathologists. False positive results associated with micropapillary pattern were also reported in the study by Turashvili (2018), where one out of two micropapillary SBTs without the *BRAF^V^*^600E^ mutation showed *BRAF* mutation-associated features [17].

Although the correlation between ECs and the *BRAF^V600E^* mutation in SBTs has been extensively studied, to our knowledge there is only one study that confirms this mutation by sequencing and detects ECs in 9% (5/46) of wild-type SBTs [8]. These results match those observed in both our previous study and the current study, in which the false positive rates were 9% (2/23) and 11% (4/36), respectively [18]. In the other works sequencing was not performed, so the *BRAF* mutation was only validated by IHC staining with the BRAF antibody (clone VE1) [10,17]. This limitation means that these results should be interpreted with caution, so further research on this topic needs to be undertaken.

## 5. Conclusions

This study may confirm the diagnostic utility of ECs in ovarian SBTs as potential predictive morphologic markers for the *BRAF^V600E^* mutation, since the pathologists were in broad agreement when identifying ECs. However, the obtained data must be interpreted with caution because of the small sample size, presence of false positive and negative results, and issues with mimics of ECs. No ECs were detected in *BRAF^Asp594Gly^*-mutated cases, highlighting their potential specificity for V600E. In future investigations, it might be possible to use ECs in H&E staining as a cost-effective, rapid screening tool to triage SBTs for molecular testing, particularly in resource-limited settings, with p16/Ki-67 increasing reliability.

In general, it seems that the revealed association between specific histological features and molecular alterations could improve diagnostic algorithms and risk stratification in patients with ovarian SBT.

## Figures and Tables

**Table 1:** Mutation variants found in borderline serous tumors.

Mutation	Coding DNA	Amino Acid	*n* (%)
*BRAF*	c.1799T>A	p.V600E	27 (67.5)
	c.1781A>G	p.Asp594Gly	3 (7.5)
*KRAS*	c.35G>T	p.Gly12Val	4 (10)
	c.35G>A	p.Gly12Asp	2 (5)
	c.183A>C	p.Gln61His	1 (2.5)
	c.34G>T	p.Gly12Cys	1 (2.5)
	c.35G>C	p.Gly12Ala	1 (2.5)
*NRAS*	c.182A>G	p.Gln61Arg	1 (2.5)

**Table 2:** Clinical features of serous borderline tumors stratified by genotype.

Characteristics		*BRAF* (*n* = 30)	*KRAS* (*n* = 9)	*NRAS* (*n* = 1)	Wild-Type (*n* = 23)	*p*-Value
Age (median, Q1–Q3)		35.0 (25.3–42.5)	49.0 (37.0–54.0)	59.0 (59.0–59.0)	38.0 (31.0–47.0)	0.16
FIGO stage	FIGO I	24 (88.9%)	3 (33.3%)	1 (100%)	9 (50%)	0.004 *
	FIGO > I	3 (11.1%)	6 (66.7%)	0	9 (50%)	
Localization	Unilateral	26 (86.7%)	5 (55.6%)	0	10 (43.5%)	0.004 *
	Bilateral	4 (13.3%)	4 (44.4%)	1 (100%)	13 (56.5%)	
Endosalpingiosis		3 (10.0%)	1 (11.1%)	0	6 (26%)	0.4
Disease-free survival	Short-term follow-up (*n* = 15)	3/7 (42.9%)	0/1	0/1	0/6	0.23
	Mid-term follow-up (*n* = 10)	0/5	1/2 (50%)	0/0	0/3	0.11

*—*p* ≤ 0.05.

**Table 3:** Pairwise agreement values (Cohen’s kappa).

	Pathologist 1	Pathologist 2	Pathologist 3
Pathologist 1		0.72	0.70
Pathologist 2			0.57
Validated ECs	0.65	0.58	0.75

## Data Availability

Data supporting the reported results can be presented upon reasonable request.

## Abbreviations

The following abbreviations are used in this manuscript:

SBT	Serous borderline tumor
LGSC	Low-grade serous carcinoma
ECs	Eosinophilic cells
RRM2	Regulatory subunit of ribonucleotide reductase M2
dNTP	Deoxyribonucleotide triphosphate
H&E	Hematoxylin and eosin
FFPE	Formalin-fixed, paraffin-embedded
CI	Confidence interval
OIS	Oncogene-induced senescence
IHC	Immunohistochemical

## Appendix A

**Table A1:** Eosinophilic cell scores in *BRAF*-mutated and wild-type for *BRAF* gene serous borderline tumors, determined by each pathologist and validated with IHC staining.

Case №	*BRAF* Mutation	p16/Ki-67-Positive ECs	Pathologist 1	Pathologist 2	Pathologist 3
1	Wild-type	presence	0	0	0
2	Wild-type	absence	0	0	0
3	p.V600E	presence	1	1	1
4	p.V600E	presence	0	0	0
5	p.V600E	presence	1	1	1
6	p.V600E	presence	0	1	1
7	Wild-type	absence	0	0	0
8	p.V600E	presence	1	1	1
9	Wild-type	absence	0	0	0
10	Wild-type	absence	0	0	0
11	p.V600E	presence	1	1	1
12	Wild-type	absence	0	0	0
13	Wild-type	absence	1	1	0
14	Wild-type	absence	0	0	0
15	Wild-type	absence	0	0	0
16	p.V600E	presence	0	1	1
17	Wild-type	presence	0	0	0
18	Wild-type	absence	0	0	0
19	p.V600E	presence	1	1	1
20	Wild-type	absence	0	0	0
21	p.V600E	absence	0	0	0
22	p.V600E	presence	1	1	1
23	p.V600E	presence	1	1	1
24	p.V600E	presence	1	1	1
25	Wild-type	absence	0	0	0
26	p.V600E	presence	0	0	1
27	Wild-type	absence	0	0	0
28	Wild-type	absence	0	0	0
29	Wild-type	absence	0	0	0
30	p.V600E	presence	1	1	0
31	p.V600E	presence	0	0	1
32	p.V600E	presence	1	1	1
33	p.V600E	presence	1	1	1
34	p.V600E	presence	1	0	1
35	Wild-type	absence	0	0	0
36	Wild-type	absence	0	0	0
37	p.V600E	presence	1	0	1
38	Wild-type	absence	0	0	0
39	p.V600E	presence	0	0	1
40	p.V600E	presence	1	1	1
41	Wild-type	absence	0	0	0
42	Wild-type	absence	0	0	0
43	Wild-type	absence	1	0	1
44	Wild-type	presence	1	0	1
45	Wild-type	absence	0	0	0
46	Wild-type	absence	0	0	1
47	p.V600E	presence	0	0	0
48	Wild-type	absence	0	0	0
49	Wild-type	absence	0	0	1
50	Wild-type	presence	1	1	1
51	p.V600E	presence	1	1	1
52	Wild-type	absence	0	1	0
53	Wild-type	absence	0	0	0
54	p.V600E	presence	1	1	1
55	p.V600E	presence	1	0	1
56	p.Asp594Gly	absence	0	0	0
57	Wild-type	absence	0	0	0
58	p.V600E	presence	1	1	1
59	p.V600E	presence	1	1	1
60	p.Asp594Gly	absence	0	0	0
61	Wild-type	absence	0	0	0
62	Wild-type	absence	0	0	0
63	p.Asp594Gly	absence	0	0	0
